# The Role of Behavioral and Psychological Interventions in Chronic Ocular Pain

**DOI:** 10.1007/s11916-026-01565-6

**Published:** 2026-09-30

**Authors:** Radhika S. Amin, Eliot N. Haddad, Raghavan Gopalakrishnan, Sara McCormick, Pavan Tankha, Sara Davin, Rony R. Sayegh

**Affiliations:** 1 Cole Eye Institute, Cleveland Clinic, 2042 East 102nd Street, Cleveland, OH 44106 USA; 2 Case Western Reserve University School of Medicine, Cleveland, OH USA; 3 Cleveland Clinic Lerner College of Medicine of Case Western Reserve University, Cleveland, OH USA; 4 Center for Neurological Restoration, Neurological Institute, Cleveland Clinic, Cleveland, OH USA; 5 Center for Spine and Pain Medicine, Neurological Institute, Cleveland Clinic, Cleveland, OH USA

**Keywords:** Chronic Ocular Pain, Dry Eye Disease, Psychological, Behavioral, Biopsychosocial, Multidisciplinary

## Abstract

**Purpose of Review:**

Chronic ocular pain (COP) is a debilitating condition in which patients experience pain in or around the eyes. It is strongly associated with other pain and psychological conditions. Management options remain limited and available ocular therapies are rarely effective, particularly in patients with central sensitization. As in other pain conditions, psychological and behavioral interventions are important, yet understudied and underutilized in the management of COP. This review explores the literature pertaining to such interventions for COP.

**Recent Findings:**

COP, while previously thought of as dry eye disease, is now accepted as a separate pain entity. It overlaps with other chronic pain conditions and is also associated with depression, anxiety, post-traumatic stress disorder, sleep disturbance, pain catastrophizing, fear avoidance, and somatic symptom burden, which can be conceptualized through a biopsychosocial model. In broader chronic pain populations, cognitive behavioral therapy, mindfulness-based interventions, acceptance and commitment therapy, pain neuroscience education, emotional awareness and expression therapy, and behavioral interventions have demonstrated benefit for pain-related distress, coping, function, and disability. However, evidence supporting such interventions for COP remains limited, and no formal investigation of psychological interventions for COP has been published to date.

**Summary:**

The reviewed literature provides supportive evidence for psychological and behavioral interventions as mechanism-based adjuncts for COP management within a biopsychosocial framework. Integrating these approaches through multidisciplinary care models emphasizing pain psychology may help address central pain contributors and functional disability. COP-specific trials are needed to define which patients will most benefit, optimal interventions, and implementation models in practice.

## Introduction

Chronic ocular pain (COP) is a multifactorial condition defined as pain perceived as originating from the eye or ocular surface that persists for three or more months [1–3]. Patients report burning, stinging, aching, itchiness, foreign body sensation, photophobia, and wind hyperalgesia, often with substantial pain-related functional impairment [4, 5]. Although symptoms of COP overlap with dry eye disease (DED), the distinction between COP and DED is clinically important [6, 7]. DED, which has a prevalence of up to 50% worldwide depending on the region and diagnostic criteria, is an ocular surface disorder defined by tear film instability and/or tear insufficiency [8–10]. In contrast, COP can develop without a clear inciting event or persist after ocular and systemic insults such as inflammation, infection, surface disease, trauma, surgery, Sjögren’s disease, and other autoimmune conditions [11–13]. Eventually, sustained nociceptive input promotes peripheral sensitization and corneal nerve dysfunction, with subsequent sensitization of trigeminal and central pain processing pathways [3, 14, 15]. COP often reveals a discordance between symptoms and ocular surface findings [16, 17]. The symptom predominant phenomenon, “pain without stain,” is especially relevant in patients with neuropathic and nociplastic pain phenotypes, in whom symptoms persist despite minimal objective signs of ocular surface disease [14, 16]. Recognizing this progression is important because treatments directed at the ocular surface are typically insufficient for patients with centrally mediated pain [3].

As in other chronic pain conditions, patient education and environmental modifications, such as reduced airflow, limited screen time, irritant avoidance, and increased humidity, are key early steps for the management of COP [6]. When neuropathic or nociplastic mechanisms contribute, further options include autologous serum tears, systemic neuromodulators, low dose naltrexone, and, in select refractory cases, trigeminal nerve stimulation, intrathecal pain pumps, botulinum toxin, or periocular nerve blocks [2, 3, 18–20]. These strategies remain essential especially for patients with active surface disease or peripheral nerve dysfunction; however, they do not fully address the cognitive, behavioral, and affective processes that sustain chronic pain. COP overlaps with other chronic pain and psychological conditions associated with central sensitization, such as fibromyalgia, migraine, irritable bowel syndrome, pelvic pain, sleep disturbance, depression, anxiety, and post-traumatic stress disorder [2, 15]. In these comorbid conditions and other chronic pain disorders, management is best informed through a biopsychosocial framework that recognizes how emotional state, sleep, attention, coping, and pain-related behaviors interact with neurobiological pain mechanisms to shape symptom persistence and functional impairment [21, 22]. This framework inherently guides evidence-based psychological interventions including cognitive behavioral therapy (CBT), mindfulness-based interventions, acceptance and commitment therapy (ACT), pain neuroscience education (PNE), emotional awareness and expression therapy (EAET), and behavioral sleep interventions, which have demonstrated benefits in pain reduction and quality of life for other pain conditions [15, 23, 24]. Patients with COP also share modifiable psychological factors with other chronic pain conditions, including catastrophizing, somatic symptom burden, fear-avoidance, threat appraisal, symptom hypervigilance, autonomic arousal, maladaptive coping, sleep disruption, and pain-related disability [25–27]. We therefore review the rationale for psychological and behavioral interventions in COP, drawing on current pathophysiologic understanding of this chronic pain condition and evidence supporting such interventions in analogous chronic pain conditions and comorbid psychological disorders.

## Nociceptive, Neuropathic, and Nociplastic Mechanisms of Chronic Ocular Pain

### Nociceptive Ocular Pain

Nociceptive pain arises from direct activation of nociceptors in response to actual or threatened non-neural tissue injury [28]. Nociceptive drivers at the ocular surface may include DED, toxic exposure, inflammation, infection, trauma, or surgery [13]. Corneal sensory afferents, including polymodal nociceptors, mechanonociceptors, and cold thermoreceptors, transmit noxious stimuli through the ophthalmic division of the trigeminal nerve (V1) [3]. Nociceptive pain typically improves after the elimination of the noxious stimuli, therefore persistence or worsening of pain despite improvement in ocular surface findings should prompt concern for neuropathic and/or nociplastic components [14].

### Neuropathic Ocular Pain

Neuropathic ocular pain results from a lesion or disease of the somatosensory nervous system, and may arise after surgery, chronic inflammation, infection, trauma, neurotrophic disease or with no clear trigger [1]. Neuropathic ocular pain is suspected when pain persists after resolution of nociceptive drivers, is out of proportion to stimuli, or occurs in the absence of noxious stimuli [1, 13]. Clinically, patients may describe burning, stinging, electric sensation, foreign body sensation, photophobia, and wind sensitivity [13]. Symptom-sign discordance, ‘pain without stain,’ is a characteristic feature; patients with more severe neuropathic-type dry eye symptoms and higher ocular pain scores often do not have worse ocular surface findings [14, 29].

Peripheral nerve damage and abnormal regeneration can sensitize corneal afferents leading to spontaneous nerve firing, reduced activation thresholds, and altered ion channel expression, contributing to the key features of allodynia and hyperalgesia [1, 30, 31]. In vivo confocal microscopy has demonstrated corneal nerve abnormalities in some patients with neuropathic ocular pain, including reduced sub-basal nerve density, increased tortuosity, beading, and microneuromas [32, 33]. Sensitization may extend to trigeminal and central pain processing pathways, which is supported by a study showing that somatosensory dysfunction at remote body sites, such as the forearm, correlates with DED-related neuropathic pain [14, 24].

### Nociplastic Ocular Pain

Nociplastic pain is characterized by altered nociception that leads to amplified and dysregulated neural signaling without evidence of ongoing tissue damage sufficient to activate peripheral nociceptors or a lesion of the somatosensory nervous system sufficient to explain the pain [14, 34, 35]. Patients may report heightened sensitivity to light, wind, temperature changes, visual tasks, or other environmental stimuli that would not normally be painful. Other key features include fatigue, sleep disturbance, cognitive symptoms, mood symptoms, or generalized sensory hypersensitivity, as seen in other chronic pain conditions with nociplastic components [2, 36, 37].

Mechanisms of nociplastic pain are incompletely understood, but involve changes in prefrontal and frontal neural circuitry from biological and social triggers based on a top-down mechanistic model and/or central sensitization from persistent peripheral input based on a bottom up-model [38, 39]. Therefore, nociceptive, neuropathic, and nociplastic components are not mutually exclusive and often coexist within the same patient. In COP, nociplastic mechanisms may be dominant in patients who have severe symptoms despite minimal ocular surface findings, limited evidence of peripheral nerve injury, centralized pain features, and comorbid pain or psychological disorders [14]. The distinction is clinically important because the dominant pain mechanism influences treatment response; patients with central sensitization are likely to have poor response to conventional ocular surface therapy, and behavioral and psychological interventions may provide benefit [15, 40].

### Understanding Pain Features Through the Biopsychosocial Model

The biopsychosocial model posits that chronic pain emerges from dynamic and reciprocal interactions among biological, psychological, and social dimensions rather than from any single biomedical mechanism [41]. Within this model, pain experience is shaped by interactions of factors from all three domains, including catastrophizing, fear-avoidance, coping, anxiety, social support, socioeconomic status, cultural context, age, sex, sleep, and hormonal influences [22]. Based on biopsychosocial theories, psychological and social factors influence the probability of developing a chronic pain condition, the severity of pain-related disability, and the success or failure of various pain treatments [22].

The biopsychosocial model is directly relevant to COP, especially in patients with neuropathic or nociplastic pain components, as it recognizes the contribution of various patient-specific factors to central sensitization. Guidelines and trials implementing the biopsychosocial model recommend a multidisciplinary and multimodal treatment approach that involves pharmacotherapy, psychotherapy, integrative treatments, and invasive procedures, supporting that behavioral and psychological interventions may be a missing piece for the management of COP [41].

## Comorbid Pain Conditions

Patients with COP, particularly those with neuropathic or nociplastic features, frequently report comorbid chronic pain conditions. These include fibromyalgia, migraine, irritable bowel syndrome, temporomandibular disorders, chronic pelvic pain, back pain, and chronic fatigue syndrome. There are also parallels between demographic risk factors for other chronic pain conditions and COP, such as female predominance and increased frequency with age [2, 42, 43].

Most studies reporting increased prevalence or burden of dry eye-like symptoms among patients with chronic pain conditions found symptom differences despite similar ocular surface parameters, suggesting contribution of neuropathic or nociplastic components [42, 44]. Similarly, a recent study of symptom-sign discordance in DED, found that higher bodily pain scores were associated with symptom-dominant discordance, a finding consistent with general altered pain processing contributing to ocular symptom burden beyond objective surface findings [16]. Furthermore, patients with a greater number of comorbid pain conditions had more severe dry eye symptoms and features of neuropathic ocular pain, suggesting a graded relationship between overall pain burden and ocular pain severity [2, 29]. Accordingly, in studies showing that higher ocular pain severity was associated with fibromyalgia, depression, anxiety, and/or migraine, patients with these conditions were less responsive to standard DED treatments [18].

The overlap between COP and systemic pain conditions is important because it supports that in some patients COP may represent one manifestation of a broader centralized pain phenotype [2]. The comorbid pain conditions are also associated with central sensitization, altered descending pain modulation, generalized hypersensitivity, sleep disturbance, and pain-related disability [15]. A recent article suggests that COP may be best understood as belonging to the category of chronic overlapping pain conditions (COPCs), which includes ten chronic pain disorders that share centrally mediated or nociplastic mechanisms [2]. Therefore, their presence should prompt clinicians to consider whether ocular symptoms are being amplified or maintained by mechanisms beyond ongoing peripheral ocular surface disease that may benefit from interventions used in other chronic pain conditions.

### Fibromyalgia

Fibromyalgia is a chronic pain condition that provides a particularly relevant model due to established nociplastic pain components and significant overlap with patients who have dry eye and ocular pain [45]. Fibromyalgia is characterized by widespread pain, sensory amplification, fatigue, sleep disturbance, and cognitive and affective symptoms, many of which overlap with features reported in patients with COP [37, 45, 46]. Approximately two-thirds of patients with fibromyalgia endorse eye pain or dry eye-like symptoms, with a noticeably reduced vision-related quality of life [45]. Studies using corneal sensitivity testing and in vivo confocal microscopy have reported ocular surface and corneal nerve abnormalities in subsets of patients with fibromyalgia-associated dry eye, including alterations in corneal sensitivity, sub-basal nerve density, Langerhans cell density, and microneuromas [47]. Updated treatment guidelines for fibromyalgia emphasize a multidisciplinary approach that includes education, exercise, psychotherapy, and acupuncture, alongside pharmacologic management [48]. Based on the overlap and shared features of patients with COP and fibromyalgia, multidisciplinary care that addresses functional restoration is likely to also provide relief in COP.

## Comorbid Psychological Disorders

Psychological comorbidities, such as depression, anxiety, post-traumatic stress disorder (PTSD), and sleep disturbance are prevalent among patients with ocular pain and dry eye-like symptoms [49]. These psychologic disorders are likely most relevant to patients with neuropathic or nociplastic pain based on studies demonstrating that the association of dry eye like symptoms with mental health disorders was independent of whether patients had objective ocular surface findings [50]. There is a bidirectional relationship between psychology and pain based on the biopsychosocial model; persistent pain contributes to poor mood, sleep, coping, and function, while psychological distress heightens pain sensitivity, attentional focus on symptoms, and pain-related disability. Overall, the influence of psychological comorbidities on the perception of pain and pain sensitivity may contribute to the development and maintenance of chronic pain [50]. Studies linking PTSD and depression to higher pain severity have suggested that mental health screening and mind-body therapies could be beneficial adjunctive treatments for dry-eye like symptoms, but this has not been pursued in literature [51].

### Depression and Anxiety

Depression and anxiety are among the most frequently studied psychological comorbidities in ocular pain and dry eye [52]. In a systematic review and meta-analysis, the prevalence of depression among DED patients was 40%, with 1.81 higher odds than controls. Similarly, the prevalence of anxiety was 39% in the DED population, with 2.32 higher odds than controls [53]. In this review, and in the DREAM study, patients with depression had higher Ocular Surface Disease Index (OSDI) scores and more ocular discomfort [54]. Other studies similarly found that dry eye symptom severity correlates with neuropathic-like ocular pain complaints, chronic pain comorbidities, and symptoms of depression and anxiety [44]. One study even reported a cumulative effect, in which having both depression and anxiety was associated with higher odds of dry eye compared to having depression alone or anxiety alone [55].

### Sleep Disorders

Poor sleep quality and decreased total sleep time are also associated with dry eye symptoms [14]. In a review article, approximately half of DED patients suffered from poor sleep, with pain acting as a key mediator [26]. Additionally, studies suggest that sleep quality and sleep latency may even mediate the relationship of dry eye symptoms with both anxiety and depression [56]. COP may interfere with sleep onset and sleep continuity, while poor sleep can increase pain sensitivity, heighten emotional reactivity, and impair coping, creating a self-reinforcing cycle [57]. These studies encourage assessment of sleep quality in patients with COP and support a role for behavioral sleep interventions, sleep hygiene counseling, and relaxation training.

## The Link Between Pain and Psychology

Chronic pain and psychological distress mutually reinforce each other through shared neurobiological pathways [41, 58]. Specific psychologic factors that contribute to chronic pain are catastrophizing, somatic symptom burden, and fear-avoidance, all of which can be seen in patients with COP [59–62]. Recognition of these pain contributors is clinically important because they are potentially modifiable. Their presence helps identify patients who may benefit from behavioral or psychological interventions.

### Pain Catastrophizing

Pain catastrophizing is a cognitive–affective response to anticipated or actual pain, comprised of three domains (helplessness, magnification, and rumination) [59]. It has been shown to markedly amplify pain and is associated with poorer outcomes, heightened pain sensitivity, and impaired functioning [60]. The few studies that have investigated pain catastrophizing in DED found that a worse Pain Catastrophizing Scale (PCS) score significantly correlated to dry eye symptom severity and neuropathic type pain. Additionally, PCS was associated with pain-related daily interference, suggesting that catastrophizing may be closely linked to pain experience in COP [25]. In general chronic pain populations, catastrophizing is associated with greater pain intensity, disability, emotional distress, and healthcare utilization, making it a modifiable target for pain psychology interventions [60].

### Somatic Symptoms Burden

Somatic symptom burden, which is heightened attention to bodily sensations, increased concern about the meaning of symptoms, and distress related to perceived bodily dysfunction, may also contribute to the impact of COP [63]. It is considered a manifestation of central sensitization that drives pain independently of peripheral pathology, supporting its relevance in patients with “pain without stain” [2, 14, 64]. In one study, patients with higher somatic symptom burden or health anxiety reported more severe dry eye-like symptoms that were independent of objective surface disease, supporting the role of somatic symptom burden in patients with neuropathic or nociplastic ocular pain [61].

The presence of somatic symptoms reflects how chronic pain, uncertainty, and prior treatment failure, can amplify distress, but does not mean that the pain is imaginary. Validated instruments, such as the PHQ-15 (Patient Health Questionnaire-15) and its abbreviated version, the SSS-8 (Somatic Symptom Scale-8), are used to measure somatic symptom burden. The SSD-12 (Somatic Symptom Disorder B-Criteria Scale) specifically captures the cognitive-emotional processing components, including symptom preoccupation, health anxiety, and symptom focus [65, 66]. Somatic symptom burden is especially relevant for COP, as is the related concept of somatosensory amplification which is defined as the tendency to experience normal bodily sensations as intense and disturbing [67]. Comprehensive multidisciplinary management can allow for appropriate assessment of these pain contributors using validated tools to guide management.

### Fear Avoidance

The cognitive-behavioral fear-avoidance model of chronic pain posits that when pain is interpreted as threatening, it triggers a cascade of fear, hypervigilance, and avoidance behavior that perpetuates a cycle of disability and worsening pain [68]. Meta-analyses indicate a robust, positive association of moderate to large magnitude between pain-related fear and disability [69]. Although fear-avoidance has been most extensively studied in musculoskeletal conditions, the model is conceptually applicable to COP, where patients may develop avoidance of environmental triggers due to anticipated pain exacerbation [70]. Fear-avoidance in COP may manifest as withdrawal from visually demanding activities, social situations, or outdoor environments, contributing to functional impairment and reduced quality of life. Photophobia, also referred to as photoallodynia, is a common complaint in up to 75% of patients with dry eye-like symptoms and significantly affects daily activities, emotional well-being, and work productivity [71, 72]. COP is associated with lower visual photosensitivity discomfort thresholds and heightened neural activation in pain-processing brain regions in response to light [73, 74]. Photophobia-related avoidance in patients with COP has parallels to fear avoidance in other pain conditions and may be modifiable with psychological interventions.

## Current Management Approaches

The management of COP begins with identification and treatment of active ocular surface disease and other nociceptive drivers. This remains essential because ongoing peripheral insults can provide persistent nociceptive input and contribute to further sensitization [14].

### Conservative Management

Conservative management and risk factor modification are key early steps for ocular pain and ocular surface disease. Literature supports the utility of patient education and complementary lifestyle-based strategies, including cardiovascular exercise, acupuncture, and omega-3 fatty acid supplementation [1, 75–77]. Patient education is important because it helps patients understand the chronic pain framework, recognize symptom triggers, and sets expectations for treatment response. These approaches are supported by the biopsychosocial model, as they can improve sleep, support overall health status, and increase self-efficacy.

Environmental modifications, including reduced airflow, limited screen time, irritant avoidance, and increased humidity, can also be helpful [78–80]. Additional strategies include regular breaks during visually demanding tasks, protective eyewear in windy or dusty environments, and limiting improper or extended contact lens use.

### Ocular Surface Optimization

Therapies for ocular surface optimization are surface lubrication, replenishment of the oily layer of the tear film, eyelid hygiene, antibiotics (e.g. azithromycin, doxycycline), anti-inflammatory eyedrops (e.g. cyclosporine, lifitegrast), punctal plugs, and blood component therapy [18, 81, 82].

Ocular surface-targeted therapies are individualized based on the disease mechanism. Lubricating therapies include preservative-free artificial tears, gels, ointments, and lipid-containing formulations. For evaporative disease or meibomian gland dysfunction, treatment may include warm compresses, lid hygiene, topical or oral antibiotics, thermal pulsation, intense pulsed light, or other lid-directed procedures. For inflammatory ocular surface disease, topical corticosteroids may be used in short courses, while steroid-sparing agents, such as cyclosporine or lifitegrast, which modulate T-cell-mediated inflammation, may be used longer-term.

Blood component therapy, such as autologous serum tears, contains growth factors that may support epithelial healing and corneal nerve regeneration and is used for patients with severe or refractory neuropathic disease. Therapeutic contact lenses, bandage contact lenses, or scleral lenses can also help by protecting the ocular surface, reducing exposure-related symptoms, and maintaining a fluid reservoir over the cornea. However, persistent pain despite appropriate ocular surface optimization should prompt consideration of neuropathic or nociplastic contributors rather than escalation of topical therapy alone.

### Systemic Pharmacotherapy

When neuropathic ocular pain is suspected, management can include systemic neuromodulators used in other pain conditions, such as gabapentin, pregabalin, tricyclic antidepressants (e.g. amitriptyline, nortriptyline), duloxetine, carbamazepine, and low-dose naltrexone, although evidence specific to COP is limited [2, 3, 18]. These medications may be most relevant for patients with burning or dysesthetic pain, photoallodynia, wind sensitivity, or comorbid chronic pain conditions. Pharmacotherapy should be individualized based on patient comorbidities, medication tolerability, and the suspected contribution of peripheral versus central pain mechanisms. Gabapentinoids may be considered for neuropathic-type pain and tricyclic antidepressants or serotonin-norepinephrine reuptake inhibitors may be useful when pain coexists with sleep disturbance or mood symptoms. Low-dose naltrexone has been described as an option for refractory pain, but there is limited evidence for its role.

### Surgical or Interventional Pain Procedures

In select refractory cases, the use of trigeminal nerve stimulation, intrathecal pain pumps, botulinum toxin, or periocular nerve blocks has been reported [19, 20, 83]. Electrical neurostimulation targets afferent fibers in the corneal pain pathway, typically to modulate trigeminal afferent signaling. Noninvasive neuromodulation approaches should be reserved for carefully selected patients and incorporated within a broader multidisciplinary care plan as there are associated risks. Intrathecal analgesic infusions of fentanyl and bupivacaine have also been described in rare cases for longer pain relief. Periocular nerve blocks may be considered in patients with cutaneous allodynia, postsurgical pain, or suspected peripheral trigeminal nerve contribution. Botulinum toxin may be relevant for patients with overlapping migraine, photophobia, or periocular muscle tension.

Depending on the underlying contributor, other interventions include punctal plug placement for tear conservation, treatment of eyelid malposition, management of trichiasis or conjunctivochalasis, amniotic membrane therapy for epithelial disease, or tarsorrhaphy in severe exposure-related disease. These interventions are most appropriate when a specific anatomic or ocular surface abnormality is identified.

### Limitations of Management Options

Centrally directed neuromodulatory pharmacotherapies and interventions can reduce pain severity and reflect a growing understanding of COP as a central pain condition [41]. However, available therapies do not fully address the cognitive, behavioral, and affective processes that sustain chronic pain. Patients with centralized pain features often experience repeated treatment failures, escalating health care utilization, frustration, and reduced trust when symptoms persist despite minimal objective findings. In this setting, continued escalation of ocular surface therapy alone or systemic pain medications may have a diminishing benefit and even contribute to worsening hypersensitivity and fear [84]. This is where psychological and behavioral interventions within a multidisciplinary care model may contribute to more comprehensive and effective care, as in other chronic pain conditions [48].

## Behavioral Interventions Studied in Ocular Disease

Behavioral interventions for dry eye-like symptoms have been studied to a limited extent [85, 86]. Evaluation of laughter exercise as a mind-body intervention found that it improved ocular surface discomfort with non-inferiority to sodium hyaluronate artificial tears [86]. This work is notable because it supports that interventions affecting mood may influence ocular discomfort. Lastly, a trial of 20 EEG neurofeedback sessions over four weeks saw that there was a medium effect on pain severity and interference across participants with neuropathic ocular pain at five weeks post intervention when comparing to baseline, however the study had four participants [87]. These early studies provide evidence that nonpharmacologic interventions can improve ocular symptoms.

## Behavioral and Psychological Interventions for Chronic Pain

Although structured psychological interventions have not been studied in COP, several approaches have demonstrated benefit in other chronic pain conditions characterized by central sensitization. Studies outlining treatment principles for nociplastic pain emphasize the importance of educating patients about the sensitized nervous system, promoting self-management, and encouraging good lifestyle habits, while positioning pharmacologic pain agents as adjuncts [15, 40]. The biopsychosocial framework and recognition of psychological pain contributors inherently guides evidence-based psychological interventions including cognitive behavioral therapy (CBT), mindfulness-based interventions, acceptance and commitment therapy (ACT), pain neuroscience education (PNE), emotional awareness and expression therapy (EAET), and behavioral sleep interventions, which have demonstrated benefits in pain reduction, function, and mood for other pain conditions. The relevance of these interventions to COP lies in the recognition that chronic pain is shaped by interactions among sensory input, central processing, emotional state, attention, behavior, sleep, and function [57, 88, 89].

### Cognitive Behavioral Therapy (CBT)

CBT has the largest evidence base among psychological therapies for chronic pain. A Cochrane review of 75 randomized controlled trials (RCTs) concluded with moderate certainty that CBT produces small but significant improvements in pain, disability, and distress compared to treatment as usual or active controls, both post-treatment and at 6–12 months follow-up [23]. The 2024 AAO Dry Eye Syndrome Preferred Practice Pattern recommends CBT as a complementary therapy for neuropathic ocular pain [90]. For COP, CBT may be particularly relevant for patients whose symptoms lead to fear of environmental triggers, reduced activity, repeated reassurance-seeking, difficulty coping with photophobia or wind sensitivity, or distress related to persistent symptoms despite treatment.

### Mindfulness-Based Stress Reduction

Mindfulness-based interventions, such as mindfulness-based stress reduction (MBSR), have also shown benefit in chronic pain with efficacy comparable to CBT. In a landmark JAMA trial, both MBSR and CBT produced greater improvement in back pain and functional limitations than usual care at 26 and 52 weeks [91]. A network meta-analysis of 68 studies found that MBSR had the most promising results for pain intensity and depression, with an optimal dosage of 8 weekly sessions of 90–120 min [88]. Mindfulness approaches train patients to observe pain and distressing sensations with less reactivity, reduce symptom-related rumination, and improve emotional regulation [88, 91]. In COP, this may benefit patients with heightened attention to ocular sensations, sensory hypervigilance, anxiety related to symptom fluctuations, or difficulty disengaging from pain.

### Acceptance and Commitment Therapy (ACT)

Acceptance and commitment therapy (ACT) is another psychological approach for chronic pain. ACT has shown medium effect sizes for pain interference, functional impairment, and depression at post-treatment, with effects maintained at 3 months [92]. For neuropathic pain specifically, ACT improved pain severity and acceptance in patients with painful diabetic neuropathy [93]. ACT focuses on psychological flexibility, acceptance of difficult internal experiences, and engagement in valued activities despite ongoing symptoms. This may be especially relevant for patients with COP who experience long-term limitations that interfere with work and social activities.

### Pain Neuroscience Education (PNE)

Pain neuroscience education (PNE) provides a complementary foundation for psychological and behavioral treatment. PNE teaches patients about peripheral sensitization, central sensitization, descending pain modulation, neuroplasticity, and the difference between pain and ongoing tissue damage [94]. A systematic review found PNE effective for improving pain, disability, and psychosocial factors in patients with central sensitization conditions, such as fibromyalgia and chronic low back pain, especially when delivered in one-on-one sessions combined with other therapeutic approaches [94]. Similarly, a RCT demonstrated that a combination of PNE with cognition-targeted motor control training was superior to traditional biomedical education plus exercise for chronic spinal pain [95]. This framework is highly relevant to COP because many patients experience severe symptoms despite minimal ocular surface findings, which can be confusing, frightening, and invalidating. PNE may improve patient trust, reduce threat appraisal, and increase willingness to engage in multidisciplinary treatment.

### Emotional Awareness and Expression Therapy (EAET)

An emerging treatment for chronic pain, emotional awareness and expression therapy (EAET), is showing promise for patients with centralized pain disorders (e.g., fibromyalgia, pelvic pain, irritable bowel syndrome) [96]. EAET uses an emotion-focused approach that emphasizes the role of the brain in pain and the importance of confronting and changing previously avoided experiences that may be emotionally charged. In a clinical trial for fibromyalgia, EAET demonstrated improvement in widespread pain, physical impairment, attention, concentration, anxiety, depression, and life satisfaction over an education control. It also was superior to CBT in reducing widespread pain and in the percentage of patients reaching at least 50% pain reduction (22.5% vs. 8.3%) [46].

### Other Behavioral interventions for Chronic Pain

Behavioral interventions used for other chronic pain conditions are also applicable to COP. Paced breathing, progressive muscle relaxation, guided imagery, biofeedback, and behavioral sleep interventions can all decrease functional impairment related to chronic pain [56, 97, 98]These strategies may be particularly useful for patients whose ocular pain is worsened by stress, poor sleep, visual task demands, or cycles of overexertion and symptom flares.

Brief pain interventions are low-burden, scalable psychological or behavioral treatments that can be delivered in a single session. They are designed to improve pain coping, reduce pain-related distress, and interrupt maladaptive pain processing without requiring extensive psychotherapy. Among these, Empowered Relief is one of the best-studied brief interventions. It is a single-session (approximately 2-hour) pain management program that has demonstrated improvements in pain catastrophizing, pain interference, and quality of life across several chronic pain populations, making it a useful model for adapting brief behavioral interventions to COP [99, 100]. Pain Reprocessing Therapy (PRT) is a relatively new, evidence-based psychological treatment developed for primary chronic pain, particularly pain maintained by maladaptive central nervous system processing rather than ongoing tissue injury [101–103]. It aims to help patients reinterpret pain as non-threatening, thereby reducing fear, hypervigilance, and central sensitization. PRT is particularly attractive for COP because it is mechanism-informed, aligns with current models of central sensitization and predictive coding.

Future trials are needed to determine which interventions are most effective, how they can be applied to COP, which patient phenotypes are most likely to benefit, and which outcomes best capture meaningful improvement. Importantly, these interventions can be individualized and integrated alongside psychological interventions.

## Discussion

Despite growing understanding of neuropathic and nociplastic mechanisms in COP, literature evaluating psychological and behavioral interventions remains limited. COP has only recently been recognized as a distinct clinical entity, therefore much existing literature studies patients under the broader umbrella of DED [104]. COP-specific studies recognize central pain mechanisms, document comorbidities, and suggest general pharmacologic or procedural management options, but there are no formal evaluations of psychological interventions [2, 14]. This narrative review synthesizes the current understanding of nociceptive, neuropathic, and nociplastic mechanisms in COP; reviews the association of ocular pain with chronic pain and psychological comorbidities; summarizes current management approaches; and described a role for behavioral and psychological interventions used in analogous chronic pain conditions. Literature supports that integrating evidence-based psychological and behavioral interventions into multidisciplinary COP care may provide a more comprehensive, mechanism-based approach for patients with high pain burden and functional impairment.

Current management approaches are essential and beneficial but likely insufficient because COP involves broader alterations in pain processing [3, 14]. This is supported by the association of COP with several chronic pain conditions and psychological comorbidities, further exemplifying the bidirectional relationship between chronic pain and psychological distress [58]. Based on the summarized existing behavioral and mind-body interventions, such laughter exercise and EEG neurofeedback, we can see that nonpharmacologic interventions are able to influence ocular symptoms and pain interference [85–87]. At the same time, interventions such as CBT, mindfulness-based approaches, ACT, PNE, relaxation training, behavioral sleep interventions, and interdisciplinary pain rehabilitation have demonstrated benefit in other chronic pain populations with overlapping mechanisms [46, 91, 92, 94]. This creates a strong rationale for the utility of behavioral and psychological interventions for COP and the need for COP-specific studies of these interventions.

Mechanistically, behavioral and psychological interventions relieve chronic pain by modifying the neural circuits that generate, amplify, and regulate pain perception. Rather than acting on peripheral nociceptive input, interventions such as CBT, mindfulness-based interventions, ACT, and PNE, alter activity within distributed brain networks involved in pain appraisal, emotion, attention, and endogenous pain modulation. Neuroimaging studies have shown that these interventions can increase engagement of the prefrontal cortex and anterior cingulate cortex, regions that exert top-down cognitive control over pain, while reducing hyperactivity in the anterior insula, amygdala, and salience network that contribute to threat learning and pain-related distress [105–108]. They also modulate functional connectivity between the default mode, salience, and executive control networks, reducing maladaptive self-referential processing and hypervigilance [106]. Importantly, behavioral interventions may enhance descending pain inhibitory pathways through the periaqueductal gray and rostroventral medulla, thereby diminishing nociceptive signal amplification at the spinal and trigeminal levels [101, 109, 110]. These neuroplastic changes underlie reductions in pain catastrophizing, fear, and negative affect, resulting in decreased pain intensity and improved function. Given the growing recognition that COP reflects central sensitization and altered brain network function, targeting these central mechanisms through behavioral interventions represents a biologically plausible strategy to complement traditional therapies and improve long-term pain outcomes.

To guide management, psychological and behavioral factors contributing to pain can be conceptualized within a biopsychosocial model framework [111]. Comprehensive care must recognize dominant pain phenotype, comorbid conditions, patient goals, and degree of functional impairment [90, 112]. This is a key first step for patients with persistent or worsening symptoms as recognition of COP as a multifactorial chronic pain condition helps clinicians validate patient experiences while also expanding the therapeutic options available to them. Patients with symptoms disproportionate to signs, incomplete response to topical anesthetic, comorbid chronic pain conditions, poor sleep, psychological disorders, somatic symptom burden, catastrophizing, fear avoidance, and substantial pain-related disability are likely to benefit from early referral to pain psychology. Recognition of psychological and sleep comorbidities should not shift attention away from careful ocular evaluation; active ocular surface disease and other ocular pain contributors should first be identified and appropriately managed. However, psychological and behavioral interventions are best framed as assistive tools that address mechanisms of chronic pain within the biopsychosocial model that conventional ocular therapies do not fully capture.

A multimodal and multidisciplinary approach amongst ophthalmology, psychology, and pain medicine, allows behavioral and psychological interventions to be introduced alongside ocular treatment rather than only after available therapies have failed [40, 111]. The manner in which psychological and behavioral interventions are introduced requires careful consideration. Many patients with COP have experienced repeated treatment failures, and referral to psychology without clear explanation could lead to patient distrust. Therefore, ophthalmologists should have an active role in explaining the neurobiological basis of COP and provide rationale for psychological interventions in a manner that prioritizes patient trust. Recognizing the pain and framing psychological interventions as scientifically studied management options may improve acceptance and engagement.

Limitations of this study should be acknowledged. Much of the current evidence draws from DED populations, which include but do not consistently distinguish patients with COP. Furthermore, the majority of studies are cross-sectional and cannot establish directionality between ocular symptoms and pain or psychological comorbidities. Lastly, implementation of psychological and behavioral interventions may be limited by real-world barriers, such as limited regional availability of pain psychologists and pain support programs. As an alternative or bridge to care, eyecare professionals can provide educational reference materials with tools for skills building, support group contact information, and relaxation strategies.

A key strength of this study is a formal review of a topic that has been mentioned in various recent studies and practice guidelines, but has not been directly addressed in the literature. A narrative review allowed us to synthesize a broad and heterogeneous body of literature that would be difficult with rigid systematic categorization. Additionally, the supportive evidence is comprehensive and based on several arguments: the presence of central sensitization, overlap with other pain conditions, comorbid psychological conditions, and the success of initial behavioral intervention. Additionally, we hypothesize how each psychological intervention used in other chronic pain conditions directly addresses contributors to COP, providing theoretical frameworks for a wide range of future studies.

## Conclusion

COP is shaped by central pain contributors that can be understood through the biopsychosocial model. Comprehensive management requires attention to chronic pain contributors and modifiable risk factors with multidisciplinary care. Psychological and behavioral interventions may serve as mechanism-based adjuncts for patients with pain amplification, impaired coping, arousal, sleep disturbance, and functional disability. Importantly, such interventions prioritize overall function and quality of life. Although direct evidence in COP remains limited, the established benefit of these approaches in comorbid chronic pain and psychological disorders provides a strong rationale for benefit in multidisciplinary COP management. Future research should determine which interventions are most effective, which patients are most likely to benefit, and how pain psychology can be integrated while preserving validation and patient trust.

## Key References

- Chang J, Hattenhauer A, Saccaro L, Mamatkazina F, Felix E, De Lott L, et al. Chronic Ocular Surface Pain: A Missing Member of the Chronic Overlapping Pain Conditions? Drugs. 2026; https://doi.org/10.1007/s40265-026-02343-9.
  - ○ This recent narrative review synthesizes evidence supporting that chronic ocular surface pain (COSP) shares epidemiologic, clinical, and mechanistic features with chronic overlapping pain conditions (COPCs). It highlights shared nociplastic pain processes and psychosocial comorbidities, providing a framework for understanding COSP beyond ocular surface pathology.
- De Lott LB, Kaplan C, Harte S, Clauw DJ, Galor A, Vehof J, et al. Nociplastic pain among individuals with chronic ocular surface pain: One cause for “pain without stain”? Surv Ophthalmol. 2025;70:536–43. https://doi.org/10.1016/j.survophthal.2025.01.004.
  - ○ This article summarizes evidence for the presence of nociplastic pain in a portion of patients with chronic ocular surface pain (COSP). The article discusses the presence of amplified and/or dysregulated neural signaling and sensory processing within the central nervous system, supporting a role for treatment approaches that extend beyond the ocular surface.

## Data Availability

No datasets were generated or analysed during the current study.

## References

[1] Ebrahimiadib N, Yousefshahi F, Abdi P, Ghahari M, Modjtahedi BS. Ocular Neuropathic Pain: An Overview Focusing on Ocular Surface Pains. Clin Ophthalmol Dove Med Press. 2020;14:2843–54. 10.2147/OPTH.S262060. PMC7524198 33061269

[2] Chang J, Hattenhauer A, Saccaro L, Mamatkazina F, Felix E, De Lott L, et al. Chronic Ocular Surface Pain: A Missing Member of the Chronic Overlapping Pain Conditions? Drugs. 2026. 10.1007/s40265-026-02343-9. PMC13375826 42271120

[3] Galor A, Hamrah P, Haque S, Attal N, Labetoulle M. Understanding chronic ocular surface pain: An unmet need for targeted drug therapy. Ocul Surf. 2022;26:148–56. 10.1016/j.jtos.2022.08.005. 35970433

[4] Morthen MK, Magno MS, Utheim TP, Snieder H, Hammond CJ, Vehof J. The physical and mental burden of dry eye disease: A large population-based study investigating the relationship with health-related quality of life and its determinants. Ocul Surf. 2021;21:107–17. 10.1016/j.jtos.2021.05.006. 34044135

[5] Andersen HH, Yosipovitch G, Galor A. Neuropathic Symptoms of the Ocular Surface: Dryness, Pain, and Itch. Curr Opin Allergy Clin Immunol. 2017;17:373–81. 10.1097/ACI.0000000000000389. PMC5995578 28858914

[6] Galor A, De Lott LB, Elsevier BV. 2025 [cited 2025 July 15]; 10.1016/j.ophtha.2025.06.015.

[7] Galor A, Abbott K, Henderson B, Pflugfelder S, White D, Gupta P, et al. Chronic Ocular Surface Pain: An Optometrist and Ophthalmologist Survey. Ophthalmol Ther. 2025;14:2801–18. 10.1007/s40123-025-01234-8. PMC12534689 40911267

[8] McCann P, Abraham AG, Mukhopadhyay A, Panagiotopoulou K, Chen H, Rittiphairoj T, et al. Prevalence and Incidence of Dry Eye and Meibomian Gland Dysfunction in the United States: A Systematic Review and Meta-analysis. JAMA Ophthalmol. 2022;140:1181–92. 10.1001/jamaophthalmol.2022.4394. PMC9614673 36301551

[9] Jones L, Craig JP, Markoulli M, Karpecki P, Akpek EK, Basu S, et al. TFOS DEWS III: Management and Therapy. Am J Ophthalmol. 2025;279:289–386. 10.1016/j.ajo.2025.05.039. 40467022

[10] Craig JP, Nelson JD, Azar DT, Belmonte C, Bron AJ, Chauhan SK, et al. TFOS DEWS II Report Executive Summary. Ocul Surf. 2017;15:802–12. 10.1016/j.jtos.2017.08.003. 28797892

[11] Ferrari G, Odorici S, Menean M, Di Zazzo A, Versura P, Micera A. The Role of Innervation in Ocular Surface Homeostasis, Tissue Remodeling Following Nerve Injury, and the Therapeutic Potential of Hemocomponents in Neuronal and Cicatricial Pathologies. J Clin Med. 2026;15:2026. 10.3390/jcm15052026. PMC12985784 41827440

[12] Graca M, Sarantopoulos K, Horn DB. Chemical toxic exposures and chronic ocular pain. Front Toxicol [Internet]. Frontiers; 2023 [cited 2026 July 4];5. 10.3389/ftox.2023.1188152 PMC10448520 37637478

[13] Mehra D, Cohen NK, Galor A. Ocular Surface Pain: A Narrative Review. Ophthalmol Ther. 2020;9:1–21. 10.1007/s40123-020-00263-9. PMC7406607 32500435

[14] De Lott LB, Kaplan C, Harte S, Clauw DJ, Galor A, Vehof J, et al. Nociplastic pain among individuals with chronic ocular surface pain: One cause for pain without stain? Surv Ophthalmol. 2025;70:536–43. 10.1016/j.survophthal.2025.01.004. PMC11884997 39814104

[15] Fitzcharles M-A, Cohen SP, Clauw DJ, Littlejohn G, Usui C, Häuser W. Nociplastic pain: towards an understanding of prevalent pain conditions. Lancet Elsevier. 2021;397:2098–110. 10.1016/S0140-6736(21)00392-5. 34062144

[16] Matar K, Yu Y, Augello P, Ying G-S, Asbell PA, Sayegh RR. Defining Discordance Between Signs and Symptoms in Dry Eye Disease: Insights From the Dry Eye Assessment and Management (DREAM) Study. Am J Ophthalmol. 2026;286:41–9. 10.1016/j.ajo.2026.02.031. 41748057

[17] Xiong F, Arnold BF, Lietman TM, Gonzales JA. Predictors of Discordance Between Dry Eye Symptoms and Signs: Insights From the Sjögren’s International Collaborative Clinical Alliance. Invest Ophthalmol Vis Sci. 2024;65:3. 10.1167/iovs.65.12.3. PMC11457922 39365264

[18] Siedlecki AN, Smith SD, Siedlecki AR, Hayek SM, Sayegh RR. Ocular pain response to treatment in dry eye patients. Ocul Surf. 2020;18:305–11. 10.1016/j.jtos.2019.12.004. 31931186

[19] Sayegh RR, Sweet JA, Miller JP, Hayek SM. Electrical Stimulation of the Trigeminal Ganglion and Intrathecal Drug Delivery Systems for the Management of Corneal Neuropathic Pain. Cornea. 2016;35:576–7. 10.1097/ICO.0000000000000751. 26807903

[20] Hayek SM, Sweet JA, Miller JP, Sayegh RR. Successful Management of Corneal Neuropathic Pain with Intrathecal Targeted Drug Delivery. Pain Med. 2016;17:1302–7. 10.1093/pm/pnv058. 26814286

[21] Raja SN, Carr DB, Cohen M, Finnerup NB, Flor H, Gibson S, et al. The revised International Association for the Study of Pain definition of pain: concepts, challenges, and compromises. Pain. 2020;161:1976–82. 10.1097/j.pain.0000000000001939. PMC7680716 32694387

[22] Meints SM, Edwards RR. Evaluating Psychosocial Contributions to Chronic Pain Outcomes. Prog Neuropsychopharmacol Biol Psychiatry. 2018;87:168–82. 10.1016/j.pnpbp.2018.01.017. PMC6067990 29408484

[23] Williams AC, de Fisher C, Hearn E, Eccleston L. C. Psychological therapies for the management of chronic pain (excluding headache) in adults - Williams, AC de C – 2020 | Cochrane Library. [cited 2026 May 24]; https://www.cochranelibrary.com/cdsr/doi/. Accessed 24 May 2026.

[24] National Institute for Health and Care Excellence (NICE). Chronic pain (primary and secondary) in over 16s: assessment of all chronic pain and management of chronic primary pain [Internet]. London; 2021. http://www.ncbi.nlm.nih.gov/books/NBK569960/. cited 2026 July 4. Accessed 4 July 2026. 33939353

[25] Patel S, Felix ER, Levitt RC, Sarantopoulos CD, Galor A. Dysfunctional Coping Mechanisms Contribute to Dry Eye Symptoms. J Clin Med. 2019;8:901. 10.3390/jcm8060901. PMC6617174 31238545

[26] Ayaki M, Tsubota K, Kawashima M, Kishimoto T, Mimura M, Negishi K. Sleep Disorders are a Prevalent and Serious Comorbidity in Dry Eye. Invest Ophthalmol Vis Sci. 2018;59:DES143–50. 10.1167/iovs.17-23467. 30481819

[27] Zhang W, Löffler M, Usai K, Mišić M, Nees F, Flor H. Hypervigilance to Pain May Predict the Transition from Subacute to Chronic Back Pain: A Longitudinal Observational Study. JPR Dove Press. 2025;18:3141–58. 10.2147/JPR.S512911. PMC12206421 40584771

[28] Terminology | International Association for the Study of Pain [Internet]. International Association for the Study of Pain (IASP). [cited 2026 July 6]. https://www.iasp-pain.org/resources/terminology/. Accessed 6 July 2026.

[29] Galor A, Covington D, Levitt AE, McManus KT, Seiden B, Felix ER, et al. Neuropathic Ocular Pain due to Dry Eye Is Associated With Multiple Comorbid Chronic Pain Syndromes. J Pain Elsevier. 2016;17:310–8. 10.1016/j.jpain.2015.10.019. PMC4775291 26606863

[30] Fakih D, Zhao Z, Nicolle P, Reboussin E, Joubert F, Luzu J, et al. Chronic dry eye induced corneal hypersensitivity, neuroinflammatory responses, and synaptic plasticity in the mouse trigeminal brainstem. J Neuroinflammation. 2019;16:268. 10.1186/s12974-019-1656-4. PMC6918709 31847868

[31] Asiedu K. Neurophysiology of corneal neuropathic pain and emerging pharmacotherapeutics. J Neurosci Res. 2024;102:e25285. 10.1002/jnr.25285. 38284865

[32] Aggarwal S, Kheirkhah A, Cavalcanti BM, Cruzat A, Colon C, Brown E, et al. Autologous Serum Tears for Treatment of Photoallodynia in Patients with Corneal Neuropathy: Efficacy and Evaluation with In Vivo Confocal Microscopy. Ocul Surf. 2015;13:250–62. 10.1016/j.jtos.2015.01.005. PMC4499014 26045233

[33] Ross AR, Al-Aqaba MA, Almaazmi A, Messina M, Nubile M, Mastropasqua L, et al. Clinical and in vivo confocal microscopic features of neuropathic corneal pain. Br J Ophthalmol BMJ Publishing Group Ltd. 2020;104:768–75. 10.1136/bjophthalmol-2019-314799. 31533927

[34] Amin RS, Haddad EN, Sayegh RR. Chronic ocular pain. Cleve Clin J Med. 2025;92:652. 10.3949/ccjm.92c.11003. 41184076

[35] Yoo Y-M, Kim K-H. Current understanding of nociplastic pain. Korean J Pain. 2024;37:107–18. 10.3344/kjp.23326. PMC10985487 38504389

[36] Kaplan CM, Kelleher E, Irani A, Schrepf A, Clauw DJ, Harte SE. Deciphering nociplastic pain: clinical features, risk factors and potential mechanisms. Nat Rev Neurol Nat Publishing Group. 2024;20:347–63. 10.1038/s41582-024-00966-8. 38755449

[37] Kelleher EM, Lange F, Wanigasekera V, Rathod-Mistry T, Nichols T, Seymour B, et al. Brain signatures of nociplastic pain: Fibromyalgia Index and descending modulation at population level. Brain. 2026;149:1365–80. 10.1093/brain/awaf307. PMC7618718 40819274

[38] Larkin TE, Kaplan CM, Schrepf A, Ichesco E, Mawla I, Harte SE, et al. Altered network architecture of functional brain communities in chronic nociplastic pain. NeuroImage. 2021;226:117504. 10.1016/j.neuroimage.2020.117504. 33293261

[39] Harte SE, Harris RE, Clauw DJ. The neurobiology of central sensitization. J Appl Biobehavioral Res. 2018;23:e12137. 10.1111/jabr.12137.

[40] Ablin JN. Nociplastic Pain: A Critical Paradigm for Multidisciplinary Recognition and Management. J Clin Med. 2024;13:5741. 10.3390/jcm13195741. PMC11476668 39407801

[41] Cohen SP, Vase L, Hooten WM. Chronic pain: an update on burden, best practices, and new advances. Lancet Lond Engl. 2021;397:2082–97. 10.1016/S0140-6736(21)00393-7. 34062143

[42] Farhangi M, Diel RJ, Buse DC, Huang AM, Levitt RC, Sarantopoulos CD, et al. Individuals with migraine have a different dry eye symptom profile than individuals without migraine. Br J Ophthalmol BMJ Publishing Group Ltd. 2020;104:260–4. 10.1136/bjophthalmol-2018-313471. 31040130

[43] Lee CJ, Levitt RC, Felix ER, Sarantopoulos CD, Galor A. Evidence that dry eye is a comorbid pain condition in a U.S. veteran population. PAIN Rep. 2:e629. 10.1097/PR9.0000000000000629 PMC5741329 29392243

[44] Chang VS, Rose TP, Karp CL, Levitt RC, Sarantopoulos C, Galor A. Neuropathic-Like Ocular Pain and Nonocular Comorbidities Correlate With Dry Eye Symptoms. Eye Contact Lens. 2018;44(Suppl 2):S307–13. 10.1097/ICL.0000000000000463. PMC5991979 29227460

[45] Schuster AK-G, Wettstein M, Gerhardt A, Eich W, Bieber C, Tesarz J. Eye Pain and Dry Eye in Patients with Fibromyalgia. Pain Med. 2018;19:2528–35. 10.1093/pm/pny045. 29554368

[46] Lumley MA, Schubiner H, Lockhart NA, Kidwell KM, Harte SE, Clauw DJ, et al. Emotional awareness and expression therapy, cognitive behavioral therapy, and education for fibromyalgia: a cluster-randomized controlled trial. Pain. 2017;158:2354–63. 10.1097/j.pain.0000000000001036. PMC5680092 28796118

[47] Emekli DT, Sahutoglu E, Öztürk GY, Kocyigit BF. Anterior segment alterations in fibromyalgia syndrome: a cross-sectional study on dry eye disease, meibomian gland dysfunction, and astigmatism. Rheumatol Int. 2025;45:120. 10.1007/s00296-025-05872-w. PMC12031864 40278912

[48] Jones EA, Asaad F, Patel N, Jain E, Abd-Elsayed A. Management of Fibromyalgia: An Update. Biomedicines. 2024;12:1266. 10.3390/biomedicines12061266. PMC11201510 38927473

[49] Yang K, Wu S, Ke L, Zhang H, Wan S, Lu M, et al. Association between potential factors and dry eye disease: A systematic review and meta-analysis. Med (Baltim). 2024;103:e41019. 10.1097/MD.0000000000041019. PMC11688006 39969375

[50] Vehof J, Kozareva D, Hysi PG, Harris J, Nessa A, Williams FK, et al. Relationship Between Dry Eye Symptoms and Pain Sensitivity. JAMA Ophthalmol. 2013;131:1304–8. 10.1001/jamaophthalmol.2013.4399. 23907167

[51] Leonardi A, Feuerman OM, Salami E, Lazzarini D, Cavarzeran F, Freo U, et al. Coexistence of neuropathic corneal pain, corneal nerve abnormalities, depression, and low quality of life. Eye (Lond). Lond Engl. 2024;38:499–506. 10.1038/s41433-023-02710-w. PMC10858266 37620513

[52] van der Vaart R, Weaver MA, Lefebvre C, Davis RM. The association between dry eye disease and depression and anxiety in a large population-based study. Am J Ophthalmol. 2015;159:470–4. 10.1016/j.ajo.2014.11.028. PMC4329250 25461298

[53] Zhao AT, He J, Lei Y, Chen Y, Ying G-S. Associations Between Dry Eye Disease and Mental Health Conditions in the All of Us Research Program. Am J Ophthalmol Elsevier. 2025;270:61–6. 10.1016/j.ajo.2024.10.009. PMC11735302 39424024

[54] Zhou Y, Murrough J, Yu Y, Roy N, Sayegh R, Asbell P, et al. Association Between Depression and Severity of Dry Eye Symptoms, Signs, and Inflammatory Markers in the DREAM Study. JAMA Ophthalmol. 2022;140:392–9. 10.1001/jamaophthalmol.2022.0140. PMC8914873 35266971

[55] Li G, Garzon C, Klawe J, Akpek EK, Ahmad S. Demographic and Psychiatric Associations With Dry Eye in a Medicare Population. Cornea. 2024;43:1499–505. 10.1097/ICO.0000000000003516. 38456830

[56] He Q, Chen Z, Xie C, Liu L, Wei R. The Association Between Dry Eye Disease With Depression, Anxiety and Sleep Disturbance During COVID-19. Front Psychiatry. 2021;12:802302. 10.3389/fpsyt.2021.802302. PMC8766963 35069294

[57] Kelleher EM, Wall A, Seymour B, Irani A. Why sleep matters in chronic pain: evidence across the lifespan. eBioMedicine. 2026;125:106158. 10.1016/j.ebiom.2026.106158. PMC12988550 41708400

[58] Vadivelu N, Kai AM, Kodumudi G, Babayan K, Fontes M, Burg MM. Pain and Psychology—A Reciprocal Relationship. Ochsner J. 2017;17:173–80. PMC5472077 28638291

[59] Quartana PJ, Campbell CM, Edwards RR. Pain catastrophizing: a critical review. Expert Rev Neurother. 2009;9:745–58. 10.1586/ern.09.34. PMC2696024 19402782

[60] Weissman-Fogel I, Sprecher E, Pud D. Effects of catastrophizing on pain perception and pain modulation. Exp Brain Res. 2008;186:79–85. 10.1007/s00221-007-1206-7. 18030455

[61] Yavrum F, Ozates M, Ozates S. An important contributing factor in dry eye disease: somatization. Int Ophthalmol. 2024;44. 10.1007/s10792-024-03133-6. 38652333

[62] Hirsh AT, George SZ, Bialosky JE, Robinson ME. Fear of pain, pain catastrophizing, and acute pain perception: relative prediction and timing of assessment. J Pain. 2008;9:806–12. 10.1016/j.jpain.2008.03.012. PMC2568976 18486557

[63] Fujii T, Oka H, Katsuhira J, Tonosu J, Kasahara S, Tanaka S, et al. Association between somatic symptom burden and health-related quality of life in people with chronic low back pain. PLOS ONE Public Libr Sci. 2018;13:e0193208. 10.1371/journal.pone.0193208. PMC5819824 29462181

[64] Crane AM, Feuer W, Felix ER, Levitt RC, McClellan AL, Sarantopoulos KD, et al. Evidence of central sensitisation in those with dry eye symptoms and neuropathic-like ocular pain complaints: incomplete response to topical anaesthesia and generalised heightened sensitivity to evoked pain. Br J Ophthalmol. 2017;101:1238–43. 10.1136/bjophthalmol-2016-309658. PMC13474410 28100479

[65] Hybelius J, Kosic A, Salomonsson S, Wachtler C, Wallert J, Nordin S, et al. Measurement Properties of the Patient Health Questionnaire–15 and Somatic Symptom Scale–8: A Systematic Review and Meta-Analysis. JAMA Netw Open. 2024;7:e2446603. 10.1001/jamanetworkopen.2024.46603. PMC11579800 39565620

[66] Toussaint A, Löwe B, Brähler E, Jordan P. The Somatic Symptom Disorder - B Criteria Scale (SSD-12): Factorial structure, validity and population-based norms. J Psychosom Res. 2017;97:9–17. 10.1016/j.jpsychores.2017.03.017. 28606504

[67] Löwe B, Toussaint A, Rosmalen JGM, Huang W-L, Burton C, Weigel A, et al. Persistent physical symptoms: definition, genesis, and management. Lancet Lond Engl. 2024;403:2649–62. 10.1016/S0140-6736(24)00623-8. 38879263

[68] Markfelder T, Pauli P. Fear of pain and pain intensity: Meta-analysis and systematic review. Psychol Bull. 2020;146:411–50. 10.1037/bul0000228. 32212745

[69] Zale EL, Lange KL, Fields SA, Ditre JW. The relation between pain-related fear and disability: a meta-analysis. J Pain. 2013;14:1019–30. 10.1016/j.jpain.2013.05.005. PMC3791167 23850095

[70] Liu H, Huang L, Yang Z, Li H, Wang Z, Peng L. Fear of Movement/(Re)Injury: An Update to Descriptive Review of the Related Measures. Front Psychol. 2021;12:696762. 10.3389/fpsyg.2021.696762. PMC8292789 34305755

[71] Schiffman RM, Walt JG, Jacobsen G, Doyle JJ, Lebovics G, Sumner W. Utility assessment among patients with dry eye disease. Ophthalmology. 2003;110:1412–9. 10.1016/S0161-6420(03)00462-7. 12867401

[72] Galor A, Feuer W, Lee DJ, Florez H, Cartar D, Pouyeh B, et al. Prevalence and Risk Factors of Dry Eye Syndrome in a United States Veterans Affairs Population. Am J Ophthalmol. 2011;152:377–e3842. 10.1016/j.ajo.2011.02.026. PMC4113967 21684522

[73] Karakoleva EV, Pondelis N, Talbert C, Aguilar MC, Gonzalez A, Rowaan C, et al. Heightened visual light sensitivity discomfort measured by the ocular photosensitivity analyzer is associated with chronic ocular pain. Ocul Surf. 2025;38:64–71. 10.1016/j.jtos.2025.06.007. PMC12231179 40543879

[74] Choudhury A, Reyes N, Galor A, Mehra D, Felix E, Moulton EA. Clinical Neuroimaging of Photophobia in Individuals With Chronic Ocular Surface Pain. Am J Ophthalmol. 2023;246:20–30. 10.1016/j.ajo.2022.09.020. PMC10964268 36223850

[75] Chen T, Feng L-Q, Jin Y. A Review of Acupuncture for the Treatment of Dry Eye Syndrome: Mechanisms, Efficacy, and Clinical Implications. Int J Gen Med. 2025;18:4647–58. 10.2147/IJGM.S526265. PMC12379998 40874238

[76] Wu X, Wei Q, Zhang W, Zhu D, Gao W, Ding N. Mechanism of periocular acupuncture in alleviating dry eye neuropathic pain via regulation of the periocular acupoint-trigeminal ganglion-ventral posteromedial thalamic nucleus pathway. Front Med (Lausanne). 2026;13:1803621. 10.3389/fmed.2026.1803621. PMC13183822 42164165

[77] Wang W-X, Ko M-L. Efficacy of Omega-3 Intake in Managing Dry Eye Disease: A Systematic Review and Meta-Analysis of Randomized Controlled Trials. J Clin Med. 2023;12:7026. 10.3390/jcm12227026. PMC10672334 38002640

[78] Narendra K, Singh SK, Deepa CK, Meghana S, Akanth KR, Manjushree M, et al. Exploring new Frontiers in dry eye Disease: Treatments, mechanisms, and diagnostic innovations a comprehensive review. Aspects Mol Med. 2025;5:100090. 10.1016/j.amolm.2025.100090.

[79] Patel S, Mittal R, Kumar N, Galor A. The environment and dry eye—manifestations, mechanisms, and more. Front Toxicol. 2023;5:1173683. 10.3389/ftox.2023.1173683. PMC10482047 37681211

[80] Dieckmann G, Goyal S, Hamrah P. Neuropathic Corneal Pain: Approaches for Management. Ophthalmology. 2017;124:S34–47. 10.1016/j.ophtha.2017.08.004. PMC5743225 29055360

[81] CORONIS symposium 2023. Scientific and clinical frontiers in ocular surface innervation - Galor – 2025 - Acta Ophthalmologica - Wiley Online Library [Internet]. [cited 2026 July 4]. https://onlinelibrary.wiley.com/doi/full/10.1111/aos.17450. Accessed 4 July 2026. 10.1111/aos.17450 PMC12069973 39891368

[82] Nortey J, Smith D, Seitzman GD, Gonzales JA. Topical Therapeutic Options in Corneal Neuropathic Pain. Front Pharmacol. 2021;12:769909. 10.3389/fphar.2021.769909. PMC8841414 35173607

[83] Mittal R, Patel S, Galor A. Alternative therapies for dry eye disease. Curr Opin Ophthalmol. 2021;32:348–61. 10.1097/ICU.0000000000000768. PMC8169641 34010229

[84] Moore E, Thibault P, Adams H, Sullivan MJL. Catastrophizing and pain-related fear predict failure to maintain treatment gains following participation in a pain rehabilitation program. Pain Rep. 2016;1:e567. 10.1097/PR9.0000000000000567. PMC5741354 29392192

[85] Xu Z, Shen J, Jiang M, Wang Y, Sun Y, Lu F, et al. A smartphone-based blink training application for alleviating dry eye signs and symptoms. NPJ Digit Med. 2025;8:703. 10.1038/s41746-025-02053-8. PMC12635133 41266739

[86] Li J, Liao Y, Zhang S-Y, Jin L, Congdon N, Fan Z, et al. Effect of laughter exercise versus 0.1% sodium hyaluronic acid on ocular surface discomfort in dry eye disease: non-inferiority randomised controlled trial. BMJ Clin Res ed. 2024;386:e080474. 10.1136/bmj-2024-080474. PMC12036614 39260878

[87] Hesam-Shariati N, Alexander L, Stapleton F, Newton-John T, Lin C-T, Zahara P, et al. The effect of an EEG neurofeedback intervention for corneal neuropathic pain: A single-case experimental design with multiple baselines. J Pain. 2025;32:105394. 10.1016/j.jpain.2025.105394. 40228689

[88] Zhu M, Wong SY-S, Zhong CC, Zeng Y, Xie L, Lee EK-P, et al. Which type and dosage of mindfulness-based interventions are most effective for chronic pain? A systematic review and network meta-analysis. J Psychosom Res. 2025;191:112061. 10.1016/j.jpsychores.2025.112061. 40010103

[89] Yang S, Chang MC. Chronic Pain: Structural and Functional Changes in Brain Structures and Associated Negative Affective States. Int J Mol Sci. 2019;20:3130. 10.3390/ijms20133130. PMC6650904 31248061

[90] Amescua G, Ahmad S, Cheung AY, Choi DS, Jhanji V, Lin A, et al. Dry Eye Syndrome Preferred Practice Pattern^®^. Ophthalmology. 2024;131:P1–49. 10.1016/j.ophtha.2023.12.041.

[91] Cherkin DC, Sherman KJ, Balderson BH, Cook AJ, Anderson ML, Hawkes RJ, et al. Effect of Mindfulness-Based Stress Reduction vs Cognitive Behavioral Therapy or Usual Care on Back Pain and Functional Limitations in Adults With Chronic Low Back Pain: A Randomized Clinical Trial. JAMA. 2016;315:1240–9. 10.1001/jama.2016.2323. PMC4914381 27002445

[92] Ye L, Li Y, Deng Q, Zhao X, Zhong L, Yang L. Acceptance and commitment therapy for patients with chronic pain: A systematic review and meta-analysis on psychological outcomes and quality of life. PLoS ONE. 2024;19:e0301226. 10.1371/journal.pone.0301226. PMC11178235 38875241

[93] Sayed D, Deer TR, Hagedorn JM, Sayed A, D’Souza RS, Lam CM, et al. A Systematic Guideline by the ASPN Workgroup on the Evidence, Education, and Treatment Algorithm for Painful Diabetic Neuropathy: SWEET. J Pain Res. 2024;17:1461–501. 10.2147/JPR.S451006. PMC11022879 38633823

[94] Lepri B, Romani D, Storari L, Barbari V. Effectiveness of Pain Neuroscience Education in Patients with Chronic Musculoskeletal Pain and Central Sensitization: A Systematic Review. Int J Environ Res Public Health. 2023;20:4098. 10.3390/ijerph20054098. PMC10001851 36901108

[95] Malfliet A, Kregel J, Coppieters I, De Pauw R, Meeus M, Roussel N, et al. Effect of Pain Neuroscience Education Combined With Cognition-Targeted Motor Control Training on Chronic Spinal Pain: A Randomized Clinical Trial. JAMA Neurol. 2018;75:808–17. 10.1001/jamaneurol.2018.0492. PMC6145763 29710099

[96] Lumley MA, Schubiner H. Emotional Awareness and Expression Therapy for Chronic Pain: Rationale, Principles and Techniques, Evidence, and Critical Review. Curr Rheumatol Rep. 2019;21:30. 10.1007/s11926-019-0829-6. PMC7309024 31123837

[97] Vambheim SM, Kyllo TM, Hegland S, Bystad M. Relaxation techniques as an intervention for chronic pain: A systematic review of randomized controlled trials. Heliyon. 2021;7:e07837. 10.1016/j.heliyon.2021.e07837. PMC8405991 34485731

[98] Toussaint L, Nguyen QA, Roettger C, Dixon K, Offenbächer M, Kohls N, et al. Effectiveness of Progressive Muscle Relaxation, Deep Breathing, and Guided Imagery in Promoting Psychological and Physiological States of Relaxation. Evid Based Complement Alternat Med. 2021;2021:5924040. 10.1155/2021/5924040. PMC8272667 34306146

[99] Darnall BD, Burns JW, Hong J, Roy A, Slater K, Poupore-King H, et al. Empowered Relief, cognitive behavioral therapy, and health education for people with chronic pain: a comparison of outcomes at 6-month Follow-up for a randomized controlled trial. PAIN Rep. 2024;9:e1116. 10.1097/PR9.0000000000001116. PMC10824382 38288134

[100] Darnall BD, Roy A, Chen AL, Ziadni MS, Keane RT, You DS, et al. Comparison of a Single-Session Pain Management Skills Intervention With a Single-Session Health Education Intervention and 8 Sessions of Cognitive Behavioral Therapy in Adults With Chronic Low Back Pain: A Randomized Clinical Trial. JAMA Netw Open. 2021;4:e2113401. 10.1001/jamanetworkopen.2021.13401. PMC8369357 34398206

[101] Ashar YK, Gordon A, Schubiner H, Uipi C, Knight K, Anderson Z, et al. Effect of Pain Reprocessing Therapy vs Placebo and Usual Care for Patients With Chronic Back Pain: A Randomized Clinical Trial. JAMA Psychiatry. 2022;79:13–23. 10.1001/jamapsychiatry.2021.2669. PMC8482298 34586357

[102] Tankha H, Lumley MA, Gordon A, Schubiner H, Uipi C, Harris J, et al. I don’t have chronic back pain anymore: Patient Experiences in Pain Reprocessing Therapy for Chronic Back Pain. J Pain. 2023;24:1582–93. 10.1016/j.jpain.2023.04.006. PMC11020878 37094744

[103] Ashar YK, Lumley MA, Perlis RH, Liston C, Gunning FM, Wager TD. Reattribution to Mind-Brain Processes and Recovery From Chronic Back Pain: A Secondary Analysis of a Randomized Clinical Trial. JAMA Netw Open. 2023;6:e2333846. 10.1001/jamanetworkopen.2023.33846. PMC10539987 37768666

[104] McCann P, Kruoch Z, Lopez S, Malli S, Qureshi R, Li T. Interventions for Dry Eye: An Overview of Systematic Reviews. JAMA Ophthalmol. 2024;142:58–74. 10.1001/jamaophthalmol.2023.5751. PMC11613798 38127364

[105] Wiech K. Deconstructing the sensation of pain: The influence of cognitive processes on pain perception. Volume 354. New York, N: Science; 2016. pp. 584–7. 10.1126/science.aaf8934. 27811269

[106] Bushnell MC, Ceko M, Low LA. Cognitive and emotional control of pain and its disruption in chronic pain. Nat Rev Neurosci. 2013;14:502–11. 10.1038/nrn3516. PMC4465351 23719569

[107] Seminowicz DA, Shpaner M, Keaser ML, Krauthamer GM, Mantegna J, Dumas JA, et al. Cognitive-behavioral therapy increases prefrontal cortex gray matter in patients with chronic pain. J Pain. 2013;14:1573–84. 10.1016/j.jpain.2013.07.020. PMC3874446 24135432

[108] Zeidan F, Grant JA, Brown CA, McHaffie JG, Coghill RC. Mindfulness meditation-related pain relief: Evidence for unique brain mechanisms in the regulation of pain. Neurosci Lett. 2012;520:165–73. 10.1016/j.neulet.2012.03.082. PMC3580050 22487846

[109] Tracey I, Mantyh PW. The cerebral signature for pain perception and its modulation. Neuron. 2007;55:377–91. 10.1016/j.neuron.2007.07.012. 17678852

[110] Zeidan F, Martucci KT, Kraft RA, Gordon NS, McHaffie JG, Coghill RC. Brain Mechanisms Supporting the Modulation of Pain by Mindfulness Meditation. J Neurosci. 2011;31:5540–8. 10.1523/JNEUROSCI.5791-10.2011. PMC3090218 21471390

[111] Marin TJ, Van Eerd D, Irvin E, Couban R, Koes BW, Malmivaara A, et al. Multidisciplinary biopsychosocial rehabilitation for subacute low back pain. Cochrane Database Syst Rev. 2017;2017:CD002193. 10.1002/14651858.CD002193.pub2. PMC6481490 28656659

[112] Nijs J, Leysen L, Vanlauwe J, Logghe T, Ickmans K, Polli A, et al. Treatment of central sensitization in patients with chronic pain: time for change? Expert Opin Pharmacother. 2019;20:1961–70. 10.1080/14656566.2019.1647166. 31355689

[113] Assessment of Somatosensory Function in Patients With Idiopathic Dry Eye Symptoms. | External Eye Disease | JAMA Ophthalmology | JAMA Network [Internet]. [cited 2026 May 24]. https://jamanetwork.com/journals/jamaophthalmology/fullarticle/2554928?utm_source=openevidence&utm_medium=referral. Accessed 24 May 2026. 10.1001/jamaophthalmol.2016.3642 27685701

